# Determination of machine‐specific tolerances using statistical process control analysis of long‐term uniform scanning proton machine QA results

**DOI:** 10.1002/acm2.12990

**Published:** 2020-08-01

**Authors:** Suresh Rana, Colton Eckert, Hardev Singh, Yuanshui Zheng, Michael Chacko, Mark Storey, John Chang

**Affiliations:** ^1^ Department of Medical Physics Oklahoma Proton Center Oklahoma City OK USA; ^2^ Department of Medical Physics Guangzhou Concord Cancer Center Guangzhou China; ^3^ Department of Radiation Oncology Oklahoma Proton Center Oklahoma City OK USA

**Keywords:** monthly QA, proton therapy, statistical analysis

## Abstract

**Purpose:**

The purpose of this study was twofold: (a) report the long‐term monthly quality assurance (QA) dosimetry results of the uniform scanning beam delivery system, and (b) derive the machine‐specific tolerances based on the statistic process control (SPC) methodology and compare them against the AAPM TG224 recommended tolerances.

**Methods:**

The Oklahoma Proton Center has four treatment rooms (TR1, TR2, TR3, and TR4) with a cyclotron and a universal nozzle. Monthly QA dosimetry results of four treatment rooms over a period of 6 yr (Feb 2014–Jan 2020) were retrieved from the QA database. The dosimetry parameters included dose output, range, flatness, and symmetry. The monthly QA results were analyzed using the SPC method, which included individuals and moving range (I‐MR) chart. The upper control limit (UCL) and lower control limit (LCL) were set at 3σ above and below the mean value, respectively.

**Results:**

The mean difference in dose output was −0.3% (2σ = ±0.9% and 3σ = ±1.3%) in TR1, 0% (2σ = ±1.4% and 3σ = ±2.1%) in TR2, −0.2% (2σ = ±1.0% and 3σ = ±1.6%) in TR3, and −0.5% (2σ = ±0.9% and 3σ = ±1.3%) in TR4. The mean flatness and symmetry differences of all beams among the four treatment rooms were within ±1.0%. The 3σ for the flatness difference ranged from ±0.5% to ±1.2%. The 3σ for the symmetry difference ranged from ±0.4% to ±1.4%. The SPC analysis showed that the 3σ for range 10 cm (R10), R16, and R22 were within ±1 mm, whereas the 3σ for R28 exceeded ±1 mm in two rooms (3σ = ±1.9 mm in TR2 and 3σ = ±1.3 mm in TR3).

**Conclusion:**

The 3σ of the dose output, flatness, and symmetry differences in all four rooms were comparable to the TG224 tolerance (±2%). For the uniform scanning system, if the measured range is compared against the requested range, it may not always be possible to achieve the range difference within ±1 mm (TG224) for all the ranges.

## 1 INTRODUCTION

Recently, task group 224 (TG224) of the American Association of Physicists in Medicine (AAPM) released the report on quality assurance (QA) of the proton machine.^1^ At present, proton centers across the world primarily use three delivery techniques: double scattering (DS), uniform scanning (US), and pencil beam scanning (PBS). The TG224 report recommends QA tests to be performed daily, weekly, monthly, and annual. Additionally, the TG224 states that “recommended tolerance limits for each of the recommended QA checks are tabulated, and are based on the literature and consensus data from the clinical proton experience of the task group members.”^1^ The TG224 tolerances can be used as guidelines for the machine QA, but due to variability in technologies and beam delivery systems among different proton therapy vendors, it is critical to determine the machine‐specific tolerances limits for the TG224 recommended parameters.^1^

Statistical process control (SPC) is one of the methods that can be used to determine the machine‐specific tolerance limit. The application of SPC to control charts allows users to assess the temporal stability of each test parameter and determine whether the various parameters of the system are in statistical control.^2^, ^3^, ^4^, ^5^, ^6^ Literature^2^, ^3^, ^4^ has reported using control limits at ±3σ for detecting meaningful changes in system performance. The SPC helps in monitoring the process using the control charts, which are used to distinguish between the common and special cause variations.^2^, ^3^, ^4^

Several studies^2^, ^3^, ^4^, ^5^, ^6^, ^7^, ^8^, ^9^, ^10^, ^11^ highlighted the importance of using SPC analysis in the radiotherapy department. For instance, Binny et al.^2^, ^7^ used the SPC to evaluate the beam output and symmetry of the linear accelerators. Shiraishi et al.^11^ and Stanley et al.^5^, ^6^ assessed the stability of image quality parameters using SPC. The authors observed the application of SPC in conventional photon therapy, but the literature on SPC analysis in proton therapy is limited. Rah et al.^3^ demonstrated the feasibility of SPC for patient‐specific QA in DS proton therapy. Rana et al.^4^ applied SPC to their daily QA results in PBS proton therapy. Both proton studies^3^, ^4^ were published prior to the publication of TG224. To date, there is no literature reporting the long‐term machine performance of the US proton delivery system. Also, SPC analysis of TG224 recommended monthly QA dosimetry of the US delivery mode is not available in the literature.

In the current study, the authors sought to (a) report the long‐term monthly QA dosimetry results of the US beam delivery system, and (b) derive the machine‐specific tolerances based on the SPC methodology and compare them against the AAPM TG224 recommended tolerances.

## 2 MATERIALS AND METHODS

### 2.A Beam delivery system

The Oklahoma Proton Center has four treatment rooms (TR1, TR2, TR3, and TR4) with a cyclotron and a universal nozzle (IBA, Louvain‐la‐Neuve, Belgium). The TR1 has a fixed horizontal beamline, TR2 and TR3 have two beamlines (30° and 90°), and TR4 is a full gantry. A detailed description of the IBA universal nozzle has been provided in the published studies.^12^, ^13^ In brief, the high‐energy proton beam is widened by the first scatter in the nozzle. The proton beam is downgraded to lower energy as it passes through the range modulator wheel. The beam is then scanned by horizontal and vertical scanning magnets in the nozzle such that a uniform dose is delivered for a rectangular scanning area. After passing through the ionization chambers, the proton beam exits the nozzle and passes through the snouts. The snouts for our delivery system are extendable. Apertures and range compensators are attached to the snout for clinical treatment.

### 2.B Monthly QA dosimetry tests and detectors

TG224 recommends four dosimetry tests for the monthly QA of US proton delivery.^1^ Tolerance for dose output, field flatness, and field symmetry is set at ±2% relative to the baseline, whereas tolerance for the distal range is ±1 mm.^1^ The monthly QA program at our center includes all four TG224 recommended parameters. Dose output is measured in water by placing a parallel‐plate (PPC05) ionization chamber (IBA Dosimetry, Schwarzenbruck, Germany) at the center of spread‐out Bragg peak (SOBP) for a proton beam that has a range (R) of 16 cm and modulation (M) of 10 cm (R16M10). The center of SOBP coincided with the isocenter. The snout position for dose output measurements was kept at 18 cm.

Field flatness and symmetry were acquired using IC Profiler (Sun Nuclear, Melbourne, FL, USA) in conjunction with the solid water for four different beams: R10M6, R16M10, R22M8, and R28M14. The detector plane was placed at the isocenter. For range measurements, the authors utilized a Zebra — a multilayer ionization chamber (MLIC) (IBA Dosimetry, Schwarzenbruck, Germany). The ranges were measured for R10, R16, R22, and R28. For both the range and profiles (flatness and symmetry) measurements, the snout was placed at 30 cm from the isocenter. For all dosimetry measurements, the aperture of 10‐cm‐circular diameter was utilized.

### 2.C Data analysis

Monthly QA dosimetry results of four treatment rooms over a period of 6 yr (Feb 2014–Jan 2020) were retrieved from the QA database. For dose output and range, the difference (∆) was calculated by comparing the results of each parameter against its baseline/expected value of the respective treatment room. For the flatness and symmetry, the results obtained from the IC profiler software were recorded in our monthly QA database without comparing against the baseline values. However, per recently published TG224, the flatness and symmetry results need to be compared against the baseline values. To follow the TG224 recommendation, the authors reanalyzed the flatness and symmetry results retrospectively by taking data from Feb 2014 as the baseline values.

The monthly QA results were then analyzed using the SPC method, which included individuals (I) and moving range (MR) chart.^3^, ^4^, ^7^ The control I chart has a central line represented by the mean value (X), whereas the upper control limit (UCL) and lower control limit (LCL) are set at 3σ above and below the mean value, respectively.^3^, ^4^, ^7^ If the measured data are within the UCL and LCL, the process is considered to be within control. If the measured data are outside the UCL and LCL, the process is said to be out of control. The UCL and LCL for the I chart are calculated using the following formula:

$$\bar{X}=\left(\sum_{i=1,k}X_{i}\right)/k.$$

$$\text{UCL}=\bar{X}+2.66\bar{R}$$

$$\text{LCL}=\bar{X}‐2.66\bar{R}$$

$$\bar{R}=\left(\sum_{i=2,k}MR_{i}\right)/\left(k‐1\right).$$

$$MR_{i}=\left|X_{i}‐X_{i‐1}\right|$$

where, k = number of data points and MR = moving range.

**Fig. 1:**
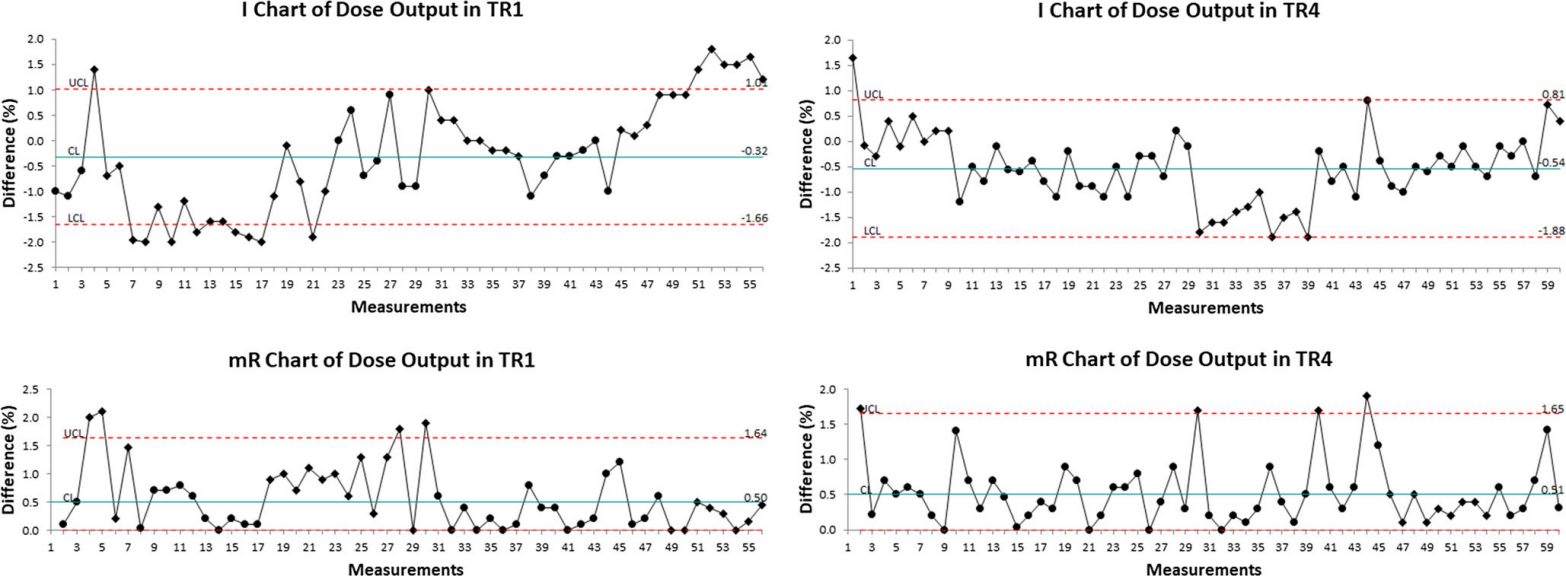
The I chart and mR chart of dose output difference in TR1 and TR4.

**Fig. 2:**
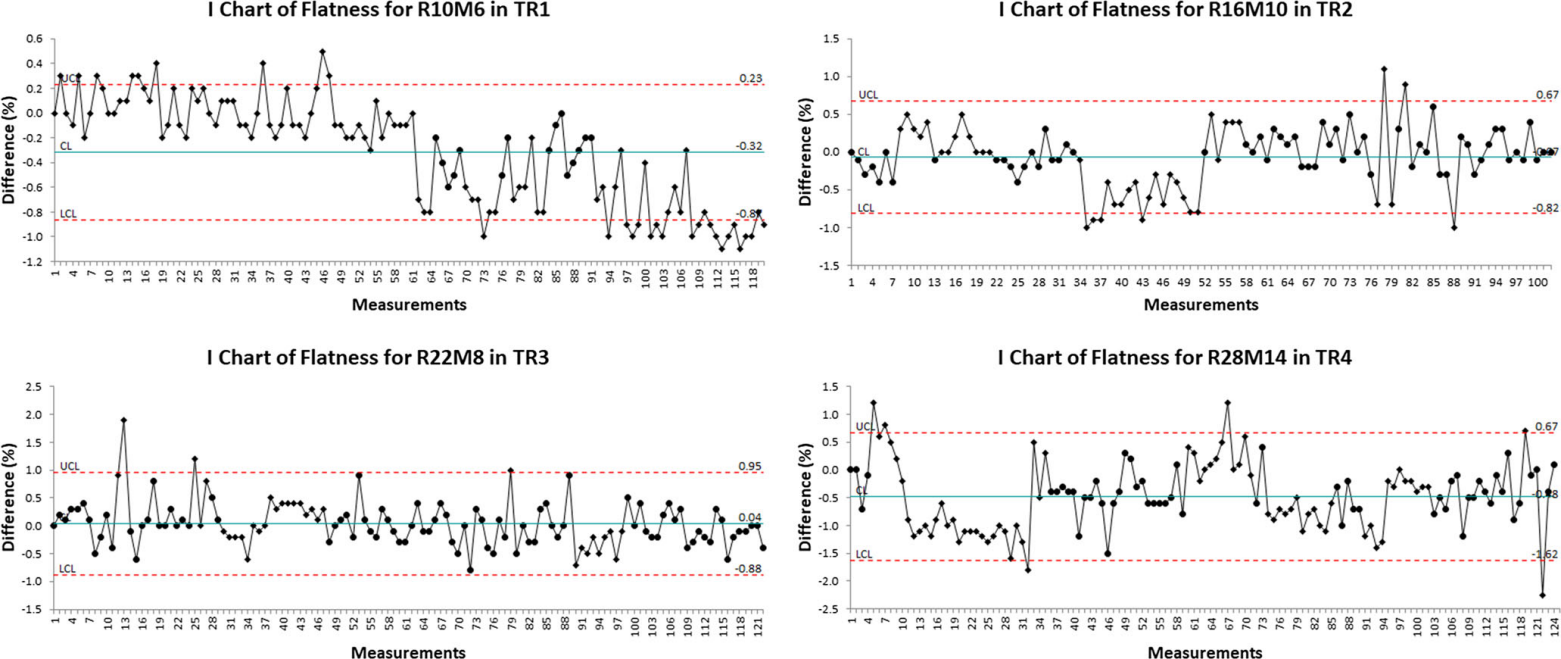
The I chart of flatness difference for selected monthly quality assurance beams in different treatment rooms.

**Fig. 3:**
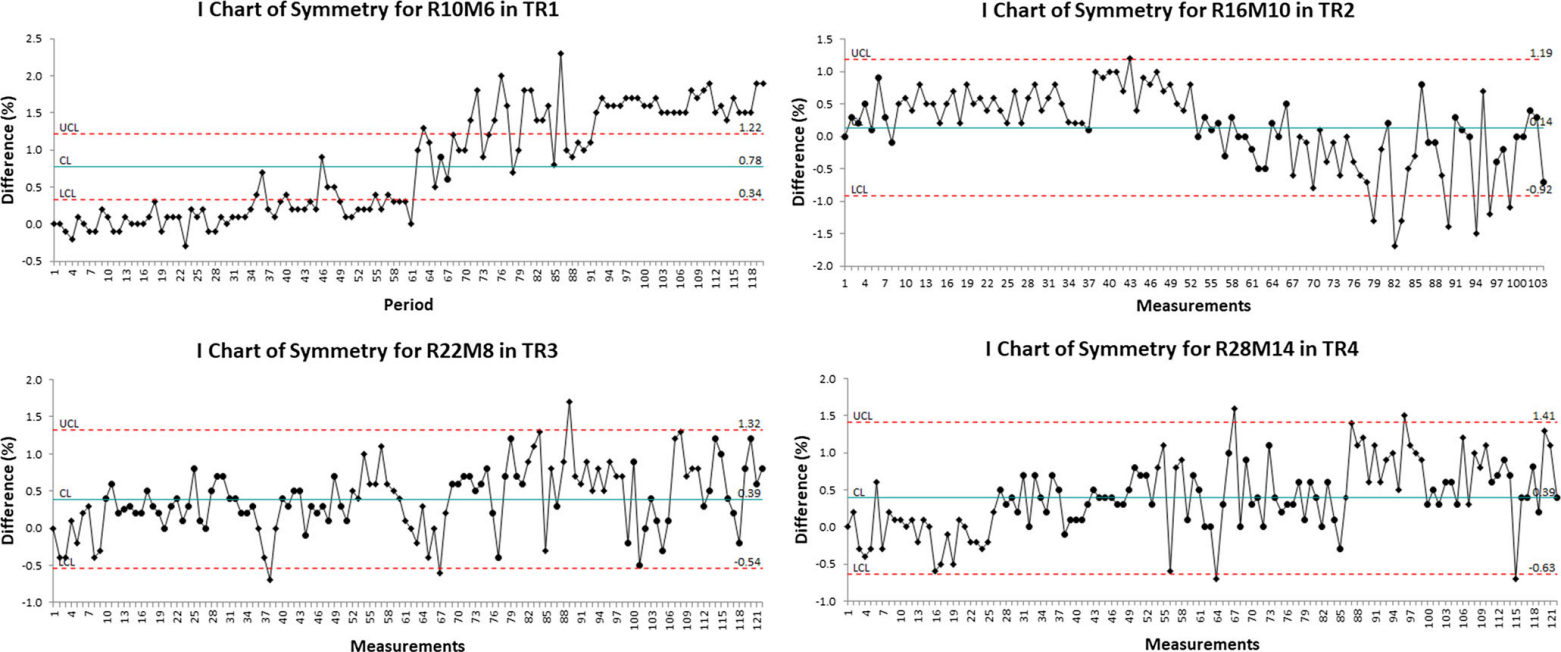
The I chart of symmetry difference for selected monthly quality assurance beams in different treatment rooms.

**Fig. 4:**
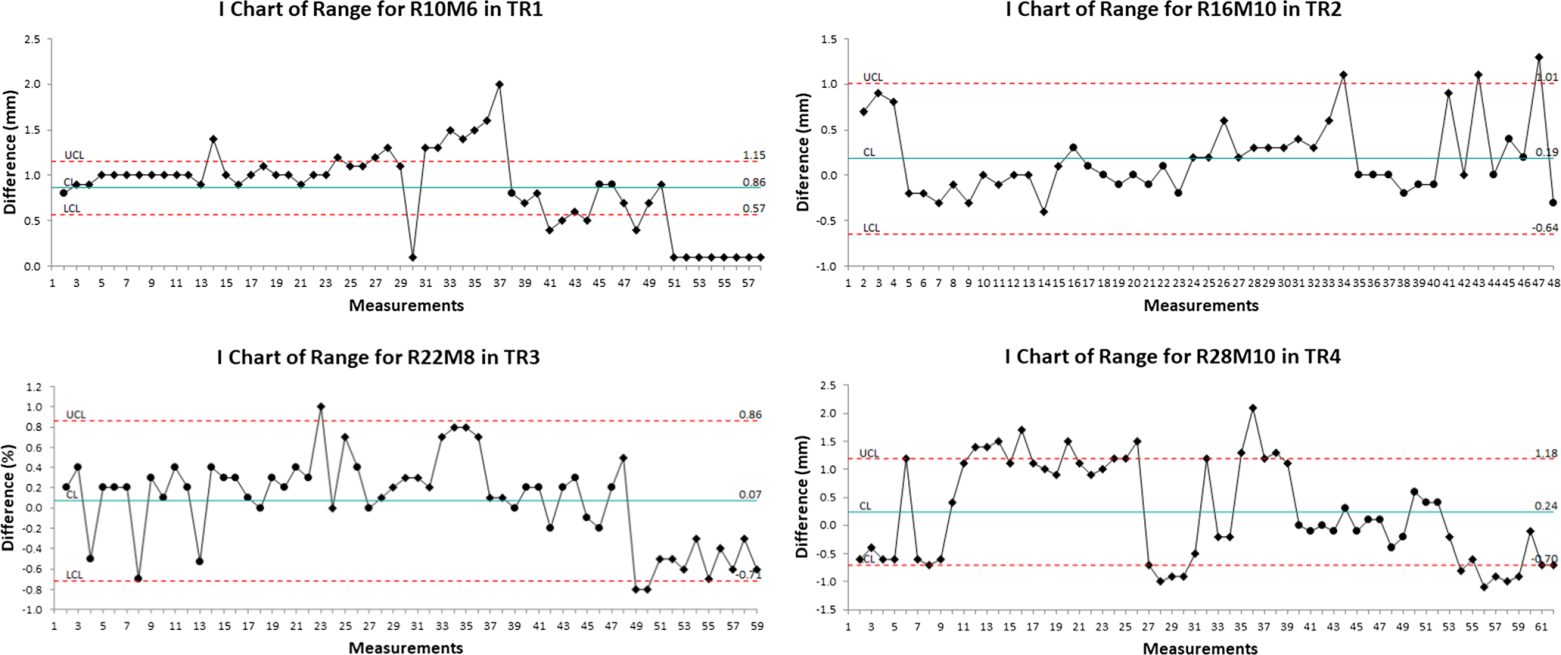
The I chart of range difference for selected monthly quality assurance beams in different treatment rooms.

Figures 1, 2, 3, 4 show the examples of the I chart that have the central line (mean) and UCL (+3σ) and LCL (−3σ) lines.

## 3 RESULTS

**Fig. 5:**
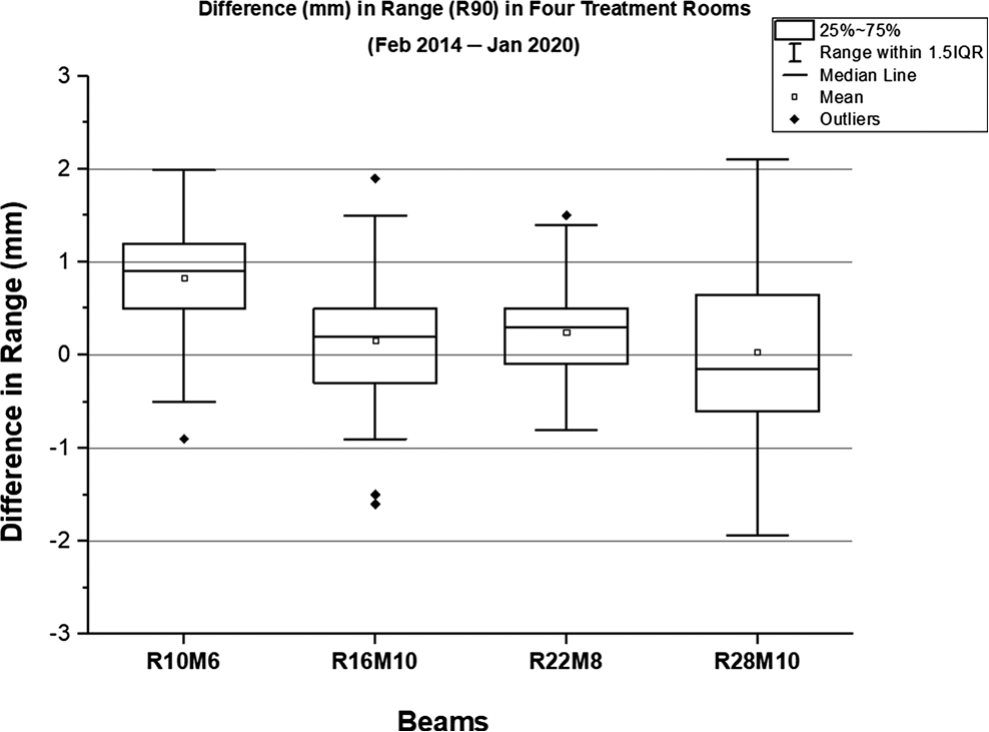
A box‐whisker plot showing the difference in range of four monthly quality assurance beams. For a given range, the results are combined from all treatment rooms.

**Fig. 6:**
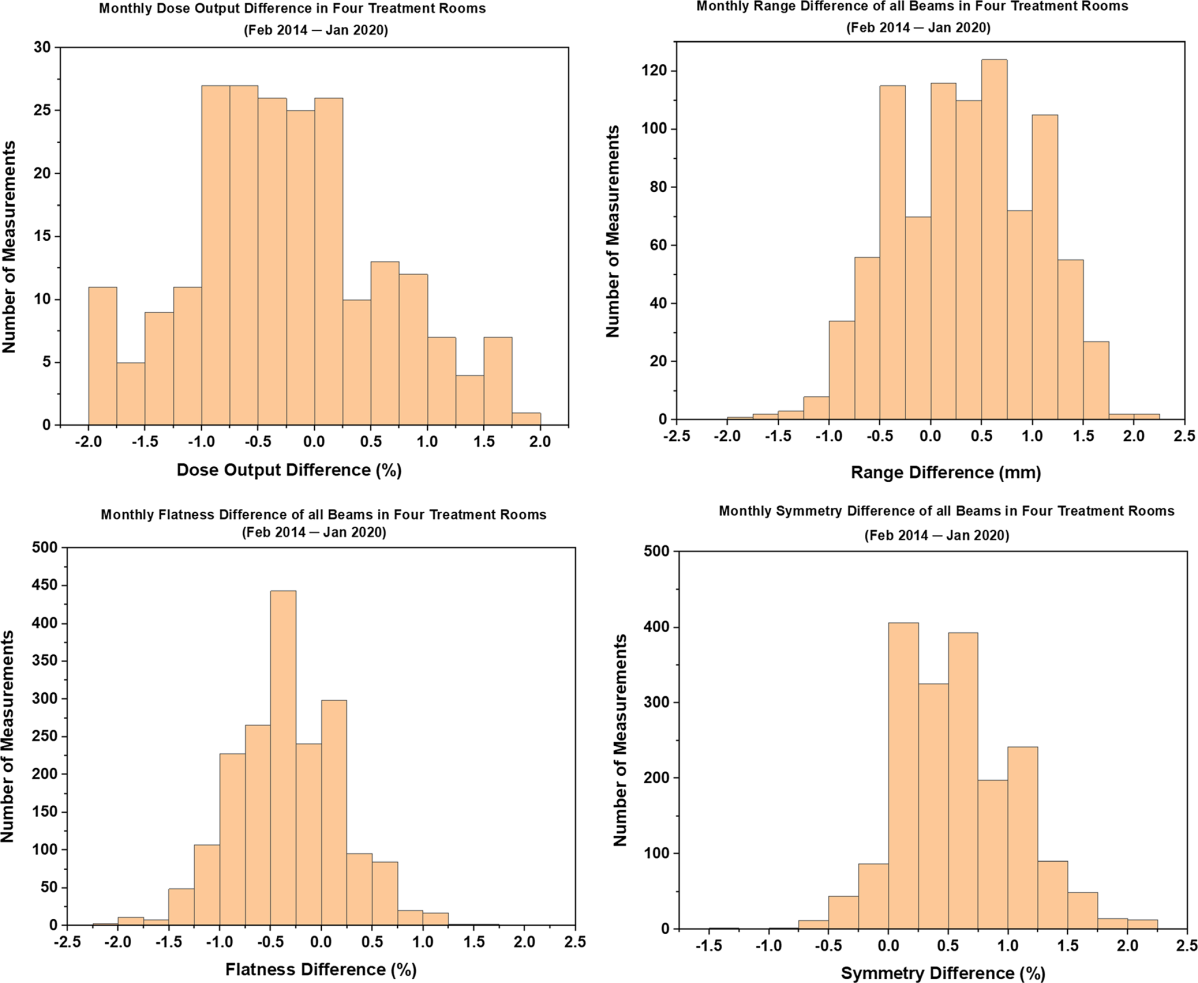
Histograms showing the difference in dose output, range, flatness, and symmetry of all monthly quality assurance beams in four treatment rooms.

Table 1 and Figs. 1, 2, 3, 4, 5, 6 show the results of monthly QA results of TG224 recommended dosimetry parameters.

**Table 1:** Monthly quality assurance (QA) results of TG224 recommended dosimetry parameters. The results included the measurements performed in four treatment rooms (TR1, TR2, TR3, and TR4) over a period of 6 yr (Feb 2014–Jan 2020). The 2σ and 3σ values were calculated using the SPC method, which included individuals and moving range control charts.

Dosimetry parameters	Mean	2σ	3σ	TG224
Dose output				
TR1	−0.3%	±0.9%	±1.3%	±2%
TR2	0.0%	±1.4%	±2.1%	±2%
TR3	−0.2%	±1.0%	±1.6%	±2%
TR4	−0.5%	±0.9%	±1.3%	±2%
Range 10 cm				
TR1	0.9 mm	±0.2 mm	±0.3 mm	±1 mm
TR2	1.0 mm	±0.2 mm	±0.3 mm	±1 mm
TR3	0.9 mm	±0.2 mm	±0.3 mm	±1 mm
TR4	0.6 mm	±0.3 mm	±0.4 mm	±1 mm
Range 16 cm				
TR1	0.2 mm	±0.3 mm	±0.4 mm	±1 mm
TR2	0.2 mm	±0.6 mm	±0.8 mm	±1 mm
TR3	−0.2 mm	±0.4 mm	±0.6 mm	±1 mm
TR4	0.4 mm	±0.3 mm	±0.4 mm	±1 mm
Range 22				
TR1	0.2 mm	±0.3 mm	±0.5 mm	±1 mm
TR2	0.5 mm	±0.3 mm	±0.5 mm	±1 mm
TR3	0.1 mm	±0.5 mm	±0.8 mm	±1 mm
TR4	0.2 mm	±0.5 mm	±0.7 mm	±1 mm
Range 28				
TR1	0.0 mm	±0.6 mm	±0.9 mm	±1 mm
TR2	0.2 mm	±1.3 mm	±1.9 mm	±1 mm
TR3	−0.3 mm	±0.9 mm	±1.4 mm	±1 mm
TR4	0.2 mm	±0.6 mm	±0.9 mm	±1 mm
Flatness R10M6				
TR1	−0.3%	±0.4%	±0.5%	±2%
TR2	−0.1%	±0.5%	±0.7%	±2%
TR3	−0.7%	±0.5%	±0.8%	±2%
TR4	−0.3%	±0.9%	±0.5%	±2%
Flatness R16M10				
TR1	−0.1%	±0.5%	±0.7%	±2%
TR2	−0.1%	±0.5%	±0.7%	±2%
TR3	0.0%	±0.4%	±0.7%	±2%
TR4	−0.6%	±0.5%	±0.8%	±2%
Flatness R22M8				
TR1	−0.1%	±0.4%	±0.6%	±2%
TR2	−0.5%	±0.6%	±0.9%	±2%
TR3	0.0%	±0.6%	±0.9%	±2%
TR4	−0.4%	±0.5%	±0.7%	±2%
Flatness R28M14				
TR1	−0.5%	±0.6%	±0.9%	±2%
TR2	−0.9%	±0.7%	±1.0%	±2%
TR3	−0.2%	±0.8%	±1.2%	±2%
TR4	−0.5%	±0.8%	±1.1%	±2%
Symmetry R10M6				
TR1	−0.8%	±0.3%	±0.4%	±2%
TR2	−0.1%	±0.5%	±0.7%	±2%
TR3	0.2%	±0.8%	±1.2%	±2%
TR4	0.4%	±0.3%	±0.5%	±2%
Symmetry R16M10				
TR1	0.0%	±0.5%	±0.8%	±2%
TR2	0.1%	±0.7%	±1.1%	±2%
TR3	−0.1%	±0.6%	±0.9%	±2%
TR4	0.7%	±0.6%	±0.9%	±2%
Symmetry R22M8				
TR1	0.5%	±0.6%	±0.8%	±2%
TR2	0.2%	±0.9%	±1.4%	±2%
TR3	0.4%	±0.6%	±0.9%	±2%
TR4	0.7%	±0.6%	±1.0%	±±2%
Symmetry R28M14				
TR1	0.3%	±0.5%	±0.7%	±2%
TR2	0.2%	±0.8%	±1.2%	±2%
TR3	0.6%	±0.7%	±1.1%	±2%
TR4	0.4%	±0.7%	±1.0%	±2%

### 3.A Dose output

The results in TR2 showed a larger spread in dose output than in other treatment rooms (TR1, TR3, and TR4). The mean difference in dose output was −0.3% (2σ = ±0.9% and 3σ = ±1.3%) in TR1, 0% (2σ = ±1.4% and 3σ = ±2.1%) in TR2, −0.2% (2σ = ±1.0% and 3σ = ±1.6%) in TR3, and −0.5% (2σ = ±0.9% and 3σ = ±1.3%) in TR4.

### 3.B Range

The ranges were evaluated for 10, 16, 22, and 28 cm. In the given treatment room, the mean difference for R10 was higher compared to the mean differences for other ranges. The evaluation of R10 among four rooms showed that TR4 produced the smallest mean difference of 0.6 mm (2σ = ±0.3 mm and 3σ = ±0.4 mm) and TR2 produced the largest mean difference of 1.0 mm (2σ = ±0.2 mm and 3σ = ±0.3 mm). Among four treatment rooms, the mean difference ranged from −0.2 to 0.4 mm for R16, from 0.1 to 0.5 mm for R22, and from −0.3 to 0.2 mm for R28. In general, the 3σ value increased with an increase in range (i.e., from R10 to R28).

### 3.C Flatness and symmetry

The mean flatness and symmetry differences of all beams among the four treatment rooms were within ±1.0%. The 3σ for the flatness difference ranged from ±0.5% to ±1.2%. The 3σ for the symmetry difference ranged from ±0.4% to ±1.4%.

## 4 DISCUSSION

The complexity of the proton beam delivery system demands rigorous QA to monitor the system performance. As there is an increasing interest in using proton therapy for cancer treatment, new vendors are joining the proton therapy market over the last decade. The uniqueness of a beam delivery system brings up a question if the QA tolerances applied on a proton machine of one vendor are applicable to the proton machine of a different vendor. Since proton therapy delivery technology is unique to the vendor, it is imperative to conduct the rigorous performance evaluation of the delivery system implemented at the institution.

The Oklahoma Proton Center (formerly known as ProCure Proton Therapy Center, Oklahoma City) started treating patients in November 2009. When the proton center performed the acceptance and commissioning of the machine in 2009, there was no consensus among the proton community regarding the tolerances that can be used as guidelines for the acceptance of the proton machine. After about a decade since the commissioning of our proton machine, the AAPM published the TG224 report for the proton machine QA in May 2019. The current study was performed with the goal of implementing the TG224 at our center. Specifically, the authors investigated the stability of various monthly QA dosimetry parameters recommended by the TG224 and developed tolerance levels based on the performance of the system.

The AAPM TG224 report does not distinguish the warning level tolerances from the action level tolerances. The inclusion of warning level tolerance can alert the user to pay close attention to the specific dosimetric parameter. The statistical analysis of dosimetry measurements over 6 yr demonstrated the feasibility of utilizing SPC results for the monthly QA tolerances. The authors believe that the 2σ values can be used as the warning level tolerances, whereas the results reaching >3σ values may require corrective action per institutional QA policies.

Currently, publications on the SPC analysis of proton machine QA results are limited. One method to characterize the machine QA process is by calculating the descriptive statistics such as the mean and standard deviation from the sample data. Such a method is effective if the random errors have a normal distribution or the sample size is large.^14^ If the sample size is large, systematic errors can be hidden in the data.^14^ Control charts, on the other hand, can identify the systematic error if it is present. Additionally, the control chart limits provide the flexibility of determining if the process is stable or unstable. If the points are within the control limits, the process can be considered stable, and these points are an indication of common cause variations. In this case, the process is in a state of statistical control. If the points are outside the control limits, the process can be considered unstable. For example, in Fig. 1, there are a few points outside the control limits, and these points are either indication of a special cause variation or false alarm. The point outside the control limit needs to be investigated to see if it still meets the institutional QA tolerance or requires the elimination of the source of a special cause variation. The current study was based on the retrospective analysis of data over a period of 6 yr. This provides the foundation to monitor the process behavior as new data points are added on the control charts of our monthly QA program.

The current dose output tolerance at our institution is ±2%, which is also a recommended value by the TG224. It was observed that the variation in dose output was worst in TR2, whereas the 2σ and 3σ results among TR1, TR3, and TR4 were in better agreement with each other. The 3σ of dose output in TR2 was slightly higher (±2.1%) than the TG224 recommendation (±2.0%). Overall, the 3σ results for the dose output in all four rooms are comparable to the TG224 tolerance.

For the flatness and symmetry, the results can be compared relative to the baseline values per TG224 report. Our monthly QA database included flatness and symmetry results, which did not require comparison against the baseline. In accordance with the TG224 guideline, the authors reanalyzed flatness and symmetry monthly QA data by comparing them against the results from February 2014. The results demonstrated that the mean difference for both the flatness and symmetry was within ±1%. The implementation of 2σ and 3σ of the flatness and symmetry for the machine QA tends to be within TG224 tolerance (±2%).

Per TG224, the distal range should be measured within ±1 mm. The report does not specify that ±1 mm tolerance is from the baseline. This means that the users are expected to compare the measured range against the requested range on the machine. Additionally, the report does not specify if the range tolerance includes detector uncertainty. Baumer et al.^15^ reported the difference within −0.1 ± 0.4 mm between Zebra measurements and measurements in a water phantom. The range data in the current study included the beams that required the input of range and modulation values in the proton beam delivery system software. The range results in the current study showed that all four beams exceeded the tolerance of ± 1 mm (TG224 recommendation) during certain months over a period of 6 yr (Feb 2014–Jan 2020). The range tolerance for the monthly QA program at our center has been set to ±1.5 mm because our proton machine was accepted with a range tolerance of ±1.5 mm in 2009. Hence, if the measured ranges are within ±1.5 mm of the requested ranges, then the vendor is not requested to resolve the range discrepancy.

The authors observed less variation in range in TR1 and TR4 than in TR2 and TR3. In TR1, the UCL and LCL ranged from 0.6 to 1.2 mm and from −0.9 to 0.6 mm, respectively. In TR4, the UCL and LCL ranged from 0.8 to 1.2 mm and from −0.7 to 0.2 mm, respectively. When comparing range deviations for the other two rooms, TR3 performed slightly better than in TR2. In TR3, the UCL and LCL ranged from 0.4 to 1.3 mm and from −1.6 to 0.6 mm, respectively. In TR2, the UCL and LCL ranged from 1.0 to 2.1 mm and from −1.7 to 0.7 mm, respectively. These results clearly suggest that it may not always be possible to achieve the range difference (i.e., without comparing against the baseline values) within ±1 mm for all ranges in our proton system; however, if the range difference is outside ±1.5 mm, the vendor is requested to fix the failing range as a part of the service agreement. The SPC analysis showed that the 3σ for R10, R16, and R22 were within ±1 mm, whereas the 3σ for R28 is exceeding ±1 mm in two rooms (3σ = ±1.9 mm in TR2 and 3σ = ±1.3 mm in TR3).

Based on the monthly QA results in the current study, the authors observed that the universal tolerance for a given metric may not always be applicable for all beams in different treatment rooms. The range accuracy could potentially differ across different rooms of the same uniform scanning delivery system. It was distinct that the dose output and range results were less consistent in TR2 compared to the results in other treatment rooms. It was also determined that TR2 exceeded the range tolerance of ±1.5 mm. At present, the patient treatment has been discontinued in TR2, and the vendor has been requested to address the dosimetry and other mechanical issues in TR2. Currently, the SPC results are used as the guidance for the recommissioning of TR2.

New proton centers are employing a PBS delivery system only. Although our study is based on the US beam delivery, the statistics from the long‐term results of our QA program can be valuable experimental information to proton centers that are employing US technique to treat proton therapy patients. The upgrade from the DS/US to PBS involves a massive financial cost and logistical challenges. The patients will continue to receive treatment in the existing proton centers that employ the US beam delivery technique. Additionally, as these existing US proton centers continue to age, the authors believe that the rigorous QA is essential to ensure optimal performance of the proton system. The authors believe that the statistical results presented in the current study will encourage other proton centers to report the QA tolerances of their proton machines from different vendors. As more proton centers start evaluating the performance of their system using SPC, the proton community will have an opportunity to further refine the proton machine QA tolerances.

## 5 CONCLUSION

The SPC analysis of dosimetry measurements over a period of 6 yr was performed to assess whether various dosimetry parameters of our USPT system were in statistical control. The authors demonstrated that the 2σ and 3σ values could be used as the warning and action level tolerances, respectively, for the monthly proton machine QA. The 3σ of the dose output, flatness, and symmetry differences in all four rooms were comparable to the TG224 tolerance (±2%). For the range, the TG224 recommends ±1 mm tolerance (not relative to the baseline values). For the USPT system, if the measured range is compared against the requested range, it may not always be possible to achieve the difference within ±1 mm for all the ranges.

## CONFLICT OF INTEREST

No conflict of interest.
